# Stem Cell Extracellular Vesicles in Skin Repair

**DOI:** 10.3390/bioengineering6010004

**Published:** 2018-12-30

**Authors:** Andrea da Fonseca Ferreira, Dawidson Assis Gomes

**Affiliations:** 1 Interdisciplinary Stem Cell Institute, Miller School of Medicine, University of Miami, 1501 NW 10th Ave, Miami, FL 33136, USA; axd1272@med.miami.edu; 2 Departamento de Bioquímica e Imunologia, Instituto de Ciências Biológicas, Universidade Federal de Minas Gerais, Av. Pres. Antônio Carlos, 6627-Pampulha, Belo Horizonte-MG 31270-901, Brazil

**Keywords:** extracellular vesicles, exosomes, stem cells, mesenchymal stem cells, skin repair

## Abstract

Stem cell extracellular vesicles (EVs) have been widely studied because of their excellent therapeutic potential. EVs from different types of stem cell can improve vascularization as well as aid in the treatment of cancer and neurodegenerative diseases. The skin is a complex organ that is susceptible to various types of injury. Strategies designed to restore epithelial tissues’ integrity with stem cell EVs have shown promising results. Different populations of stem cell EVs are able to control inflammation, accelerate skin cell migration and proliferation, control wound scarring, improve angiogenesis, and even ameliorate signs of skin aging. However, large-scale production of such stem cell EVs for human therapy is still a challenge. This review focuses on recent studies that explore the potential of stem cell EVs in skin wound healing and skin rejuvenation, as well as challenges of their use in therapy.

## 1. Introduction

Stem cells have attracted great interest from the scientific community since their discovery by Till and McCulloch in 1961 [1]. Their capacity to differentiate into various cell types and hence provide tissue repair made them promising tools in the treatment of such pathologies as neurodegenerative disorders, organ failure, and tissue damage. However, stem cells such as mesenchymal stem/stromal cells (MSCs) exert their functions via paracrine effects and not by the replacement of dead cells [2,3,4].

The term secretome refers to the complex mixture of factors released by virtually all cell types, including stem cells, to the extracellular space. Once released by stem cells, this combination of different classes of molecules can modify microenvironments by controlling inflammation as well as inducing selective protein activation and transcription. This secreted milieu of molecules may culminate in tissue regeneration [5,6,7]. Recent evidence about this paracrine mechanism has opened up a new paradigm in stem cell therapy and stimulated the search for strategies that explore the concept of “cell therapy without cells” [8,9].

The secretome of stem cells comprises lipids, proteins, and nucleic acids. Although the classes of molecules present specifically in the secretome of MSCs are similar to those found in other cell types, their therapeutic potential is unique [10,11]. The most well-studied and dynamic part of the growing field of secretomics is extracellular vesicles (EVs).

EVs represent an important fraction of virtually any cell type’s secretome [12]. Extensive research is currently being conducted to elucidate the healing potential of stem cell EVs in numerous disease processes. EVs released by stem cells to the extracellular space have been shown to improve vascularization, immunomodulation, cardiac and central nervous system regeneration, and even potentially aid cancer therapies [13,14,15,16,17].

In this review, we focus on the work that has been conducted using EVs from stem cells in skin wound healing, including their potential in skin cell proliferation, migration, angiogenesis, and the reduction of scarring. We also address limitations to the use of stem cell EVs in skin therapy.

## 2. EVs

The broad term EVs is categorized into three major classes of lipid vesicle: ectosomes, exosomes, and apoptotic bodies. This classification is based on the vesicles’ biogenesis and also relies on their difference in diameter size. It is important to note that reports somewhat vary on vesicle size classification. Ectosomes (or microvesicles) result from protrusions of the plasma membrane that eventually detach and are shed in the extracellular space, and their diameter ranges between 50 and 500 nm. Apoptotic bodies are a product of apoptosis and contain the biomaterial from the dying cell. Their size ranges from 50 to 5000 nm. The last and potentially most exciting category of EVs are exosomes. These are the smallest EVs, with a diameter that ranges from 50 to 150 nm and are born from larger intracellular vesicles called multivesicle bodies (MVBs). MVBs are intraluminal vesicles, formed by internal budding of the endosomal membrane. MVBs migrate toward the edge of the cell, where they fuse with the plasma membrane. Exosomes are then released to the extracellular space via exocytosis. This process is regulated by tumor protein p53 (p53) and under the control of the cytoskeleton activation pathway, but not affected by calcium. Exosomes contain large amounts of annexins, tetraspanins such as CD63, CD81, and CD9, and heat-shock proteins, including Hsp60, Hsp70, and Hsp90. They also express programmed cell death 6 interacting protein (Alix/PDCD6IP), tumor susceptibility gene 101 (Tsg101), and clathrin. Exosomes are encapsulated in a rigid bilayer membrane that protects their contents and enables them to move long distances in tissues. The bilayer membrane possesses small amounts of phosphatidylserine but large amounts of cholesterol, ceramide, and sphingolipids [18,19].

New strategies to isolate and purify subclasses of EVs in an efficient manner have been the subject of research of numerous groups. Examples of techniques used for this purpose are ultrafiltration, consecutive centrifugations and ultracentrifugations [20], size exclusion chromatography [21], precipitation, and immunoaffinity purification using different kits [22]. Unfortunately, it is widely accepted that none of the current methods for EVs isolation can effectively purify one class from the other. Moreover, the search for definitive exclusive biomarkers for each subtype is challenging. Hence, the International Society for EVs (ISEV) proposes the use of the broad term “EVs”, since it is likely that the scientific community is working with mixtures of subtypes [23]. However, it is common to find still the term “exosomes” used in the literature. Most preparations could be considered “exosome-enriched fractions” of EVs [24,25].

It is imperative when evaluating the literature on EVs to remember that the variation of EVs isolation and purification techniques generates different populations and, thus, may lead to distinct results. There is still a need for the standardization of laboratory techniques by the ISEV in the research conducted in the field of EVs, especially when considering the translation of experimental findings to clinical applications [23]. The most recent report from the ISEV stated that each preparation of EVs must (1) include the source of EVs defined by quantitative measures (e.g., number of secreting cells, volume of biofluid, mass of tissue); (2) be characterized to the greatest extent possible to determine the abundance of EVs (total particle number and/or protein or lipid content); (3) be tested for the presence of components associated with EVs subtypes or EVs generically, depending on the specificity one wishes to achieve; and (4) be tested for the presence of non-vesicular, co-isolated components [26].

### Stem Cell EVs

Using EVs as therapeutic tools instead of actual cells is not only an elegant approach to stem cell therapy, but it also remedies some of the concerns regarding limitations and adverse effects of earlier strategies [27]. Limitations of stem cell administration include their proliferation capacity, lifespan, and potential contamination by handling [28]. It was previously reported that injected stem cells in a given tissue or organ could cause vascular occlusion, undesired inflammatory response, human pathogen transmission, heart arrhythmia, and even tumor formation [29,30,31]. Rejection, especially in allogenic stem cell administration, is also a concern [32]. The properties of stem cell EVs do not cause these deleterious effects, making them even more appealing [33].

Stem cell EVs hinder therapeutic effects due to their unique cargo provided by their parental stem cells. Growth factors, microRNAs, long non-coding RNAs (long-ncRNAs), and various other classes of molecules present in these specific EVs populations favor tissue repair [34,35].

## 3. EVs Cargo

The elucidation of EVs’ cargo is essential to understand the mechanisms by which their therapeutic effects operate [36]. Different authors have performed analyses of stem cell EVs populations from various origins. Their work is summarized in Table 1 (we excluded papers that addressed the cargo of cancer stem cells).

Several classes of molecule are part of EVs’ cargo: cytokines, growth and transcription factors, soluble receptors, DNA, microRNAs, messenger RNAs (mRNAs), long-ncRNAs, and circular RNAs (circRNAs) [46]. Proteomic and transcriptome analyses suggest that variables such as culture media, the age of the donor (animal or human), ionizing radiation, the number of cell passages, and the method of vesicle isolation may interfere with the characteristics and content of EVs [47,48]. The number of passages in culture play an important role as well since senescent cells have been shown to release more EVs than young cells. This is perhaps due to a purging system, as EVs could be used to remove harmful molecules from cells [49]. Interestingly, it is even possible to manipulate some of these variables as a way of inducing a particular characteristic of the cargo of stem cell EVs. For example, cells subjected to hypoxia produce EVs with higher angiogenic capacity than cells that were grown in normal conditions [50]. Moreover, there is considerable cargo heterogeneity between the vesicles present in the same sample [18,19]. The mechanisms responsible for RNA packing into exosomes, for example, are intricate and rely on RNA-binding proteins (RBPs). These may form RNA-ribonucleoprotein complexes, which could be important for the transport of RNAs into exosomes and the maintenance of RNAs inside exosomes. This last effect results from the stability provided by the complex formation [51]. Although EVs’ precise cargo differs among the data that has been produced in recent years, key elements of their resulting overall function seem to be somewhat common. Many molecules, proteins, and nucleic acids could participate in processes that favor tissue regeneration, such as angiogenesis, cell migration, and the proliferation or regulation of inflammatory response [52,53,54].

## 4. Skin Wound Healing

The occurrence of skin injury is highly prevalent. It can result, for example, from trauma or surgical incision in the case of acute wounds [54]. Chronic skin wounds, on the other hand, are common comorbidity of diabetes, a complex disease that affects 30.3 million people in the United States alone [55]. Estimates state that diabetic ulcer management costs the American health system US$ 13 billion per year [56]. Skin wound healing is a complex and fine-tuned process that is broadly divided into three phases: the inflammatory, proliferative, and remodeling phases.

The first phase is marked by cytokine activity and the coagulation cascade with the recruitment of cells responsible for conducting the debridement of the tissue. Neutrophils are the most important cells early in this phase, responsible for phagocytosis and cytokines release to induce inflammation. In the late stages of the inflammatory phase, macrophages become the dominant cell type and act to control inflammation. This is why the normal function of macrophages is key to maintain homeostasis in the wound area [57]. The proliferative phase starts four days after wounding and ends around day 20, marked by migration, proliferation, extracellular matrix deposition, and angiogenesis. Transition to the next phase is essential for the normal closure of the wound and does not occur properly in chronic wounds. The third and final phase is characterized by wound contraction and collagen remodeling. Abnormal collagen deposition in this phase causes scarring abnormalities such as keloids [58,59].

Stem cell EVs have the potential to optimize all three phases of wound healing due to their capacity to control inflammation, stimulate cell migration and proliferation, and even improve scarring [60]. The work that has been done so far to elucidate mechanisms that explain the effect of stem cell EVs in wound healing usually rely on the vertical transfer of microRNAs or proteins to skin cells [61,62]. Researchers explored the regulation of well-known pathways by stem cell EVs cargo. Diverse approaches have been used to evaluate these premises, as shown in Table 1.

### 4.1. Effect of Stem Cell EVs on Skin Cells during Wound Healing Phases

#### 4.1.1. Inflammatory Phase

As mentioned above, inflammation is part of the normal wound healing course, but in the case of burn wounds or chronic wounds, this response is abnormally sustained [56]. It is known that stem cells can modulate inflammation [63] and recently it was proposed that stem cell EVs have similar properties [63,64]. Human umbilical cord MSCs (hucMSC)-derived EVs, for example, promote the significant switching of recipient macrophages (human monocytic cell line THP-1) toward the anti-inflammatory M2 phenotype. They also regulate the activation, differentiation, and proliferation of B lymphocytes and can suppress T-lymphocyte proliferation. Moreover, MSCs-derived EVs can convert activated T lymphocytes into the T-regulatory phenotype, thereby exerting immunosuppressive effects [65].

HucMSC pretreated with lipopolysaccharide (LPS) release EVs enriched with microRNA let7b. When internalized, let7b can regulate macrophage polarization by the inhibition of the Toll-like receptor 4 (TLR4)/nuclear factor-κB pathway and by activating the signal transducer and activator of transcription 3 (STAT3)/AKT serine/threonine kinase (AKT). This would resolve chronic inflammation [65].

EVs from umbilical cord stem cells can attenuate burn-induced inflammation. Li and colleagues [66] found that burn injury stimulated an inflammatory reaction in macrophages exposed to LPS, with higher production of TNF-α and IL-1β accompanied by decreased IL-10 levels. EVs from umbilical cord MSCs successfully reversed this reaction. The mechanism would result from miR-181c transfer by EVs to macrophages with a consequent suppression of the TLR4 signaling pathway and inflammatory response alleviation [66].

Moreover, an interesting study conducted by Kou and collaborators showed that TNF-α promotes EVs-IL-1RA exocytosis from MSCs, mediated via Fas/Fap-1/Cav-1. This mechanism would be more pronounced in stem cell EVs from gingiva compared to their bone marrow or skin stem cell counterparts. As IL-1RA is an antagonist to IL-1B, this could control inflammation in microenvironments [45].

#### 4.1.2. Proliferative Phase

Most of the research conducted on stem cell EVs in wound healing has focused on the second phase of wound healing, the proliferative phase. Different reports have suggested that stem cell EVs are promising tools to accelerate this step since they can improve migration and proliferation in skin cells, such as fibroblasts and keratinocytes [52,53]. Moreover, collagen and elastin deposition can be improved by stem cell EVs treatment. Various papers rely on in vitro models to elucidate the molecular mechanisms that could explain the effects of stem cell EVs.

Cell functions that take part in the proliferative phase are intricate and achieved by different mechanisms; it is likely that stem cell EVs act in diverse pathways to generate their effects. Our group previously reported that EVs from human adipose-derived MSCs could accelerate the migration and proliferation of dermal fibroblasts and keratinocytes as well as activate the AKT pathway [53]. Choi and colleagues found similar results in 2018 [67]. In fact, EVs from adipose-derived MSCs appear to optimize the characteristics of dermal fibroblasts in a dose-dependent manner. Fibroblasts that internalized those EVs showed higher expression of cyclin-1, N-cadherin, PCNA, and collagens I, III [60]. MALAT1 seems to be an important regulator of the effect of adipose-derived MSCs-EVs, as purified MALAT1-carrying EVs were able to induce skin fibroblast migration at a similar pace as FGF-2, and its depletion abolished this effect [68].

Other types of MSCs-EVs have been shown to produce the same beneficial effects. McBride and colleagues reported that bone marrow stem cell EVs can accelerate dermal fibroblast migration and proliferation as well as induce angiogenesis. These effects are further improved if CD63^+^ Wnt3a-expressing EVs are purified and used to treat skin cells. This would ultimately activate the Wnt pathway more efficiently and refine the effect of EVs for this particular purpose [69]. Human umbilical cord stem cell EVs were capable of inducing proliferation and reducing heat stress inducing-apoptosis in HaCAT and dermal fibroblasts with the activation of AKT [70]. Also, once infused in a second-degree burn injury model in rats, improved reepithelization and higher collagen I and elastin expression were observed. EVs used in this study were found to carry Wnt4, which could activate beta-catenin. When translocated to the nucleus, beta-catenin promoted fibroblast migration and proliferation. Further studies revealed that hucMSC-exosomal 14-3-3ζ mediated the binding of YAP and p-LATS by forming a complex to promote the phosphorylation of YAP, which orchestrated an exosomal Wnt4 signal in cutaneous regeneration [71]. YAP activities and phosphorylation at the Ser127 site were required for the binding of YAP and p-LATS. Also, 14-3-3ζ recruits YAP and p-LATS to form a complex under a high cell density status and 14-3-3ζ, besides YAP or p-LATS, was the key regulatory molecule of this complex. These findings collectively indicate that hucMSC-Ex functions not only as an “accelerator” of the Wnt/beta-catenin signal to repair damaged skin tissue but also as a “brake” of the signal by modulating YAP to orchestrate controlled cutaneous regeneration [71].

Kim and colleagues reported similar findings in 2017. A human growth factor antibody array was conducted in EVs from human umbilical cord stem cells. Results showed that EVs carry various growth factors that stimulate fibroblast growth and migration as well as collagen and elastin synthesis, especially EGF and bFGF. It was also demonstrated that EVs from umbilical cord stem cells could reach the human epidermis after 18 h of administration in an elegant ex-vivo model [44].

Another interesting study conducted by Tooi and collaborators showed that EVs isolated from placenta stem cells improved dermal human fibroblasts plasticity. Those skin cells had increased expression of NANOG and Oct4, both markers for stemness. More importantly, dermal fibroblasts exposed to EVs from placenta stem cells displayed competent adipocyte and osteocyte differentiation [72].

#### 4.1.3. Remodeling Phase

Normal scarring is a part of the remodeling and final phase of wound healing. Excessive scarring, however, is the result of abnormal collagen production by myofibroblasts and loss of skin function. Reducing scar formation is an interesting feature of stem cell EVs. Human amniotic stem cell EVs are also capable of reducing scarring by controlling the deposition of the extracellular matrix. Zhao and collaborators suggested that this could be partly achieved by the stimulation of matrix metalloproteinase-1 (MMP-1). Furthermore, the authors found that the organization of fibers is also improved by EVs, as they seem less organized, with a closer morphology to healthy skin [73]. Fang and colleagues proposed that umbilical cord stem cell EVs could inhibit myofibroblast differentiation by inhibiting the transforming growth factor-B/SMAD2 pathway in a mouse model. MicroRNAs miR-21, -23a, -125b, and -145 present in those EVs’ cargo would be responsible for this effect [60].

Intravenous injection of adipose-derived MSCs-EVs decreased the size of scars and prevented the differentiation of fibroblasts into myofibroblasts in addition to increasing the ratio of transforming growth factor-β3 (TGF-β3) to TGF-β1. Additionally, EVs increased the matrix metalloproteinases-3 (MMP3) expression of skin dermal fibroblasts by activating the extracellular-signal-regulated kinase (ERK)/mitogen activated protein kinase (MAPK) pathway, leading to a high ratio of MMP3 to tissue inhibitor of matrix metalloproteinases-1 (TIMP1), which is also beneficial for the remodeling of the extracellular matrix [74].

Hu and colleagues defended the interesting premise that at early stages of wound healing, those EVs induce the expression of collagen but, at late stages, EVs shift their effect towards collagen expression inhibition. That could contribute to less scarring [60].

### 4.2. Effects of Stem Cell EVs on Wound Healing

Emerging studies are evaluating stem cell EVs potential in various animal models of wound healing. Rat and mouse are usually the most used in vivo models, even if it is well known that porcine skins show a closer resemblance to human skin regarding traction and pace of healing. Common experimental strategies include the topical application or subcutaneous injection of EVs. When present, the evaluation of wound reepithelization criteria varies between studies, particularly in time points or method for histological assessment. Full-thickness excisional wounds and excisional splinting wound healing models are frequently used for the verification of skin wound area closure after administration of stem cell EVs. The preferred time points for evaluation are 7, 14, and 21 days with differing wound sizes used [53,75,76].

Healthy animals are used as models for acute wounds and diabetic mice (db/db or streptozotocin-treated mice, traditionally) are employed in chronic wound studies [76]. In vivo studies in healthy animals revealed that human adipose-derived MSCs-EVs could accelerate wound closure and scar formation with the activation of the ERK/MAPK pathway and the modulation of metalloproteinases in mice [74]. Recently, it was reported by Pelizzo and colleagues that rabbit stem cell EVs from two origins, adipose and bone marrow, were superior to stem cell injection for wound healing in vivo. The authors also stated that adipose stem cell EVs increased wound closure more efficiently than their bone marrow counterparts [75].

Although largely used, diabetic mice models for chronic wound healing are controversial. It is accepted that the key features of human chronic wounds are not mimicked by diabetic mice or rats, and this premise challenges the translation of experimental findings to patients. In fact, diabetic murine wounds display a mere delay in wound resolution but eventually heal completely [77,78]. Regardless of the major limitations of these models for human chronic wounds translation, it is important to consider studies that rely on diabetic mouse and rat wounds as they show the ability of stem cell EVs to ameliorate somewhat impaired wound healing processes.

Experimental data indicate that human endothelial progenitor cells EVs from umbilical cord stem cells accelerate cutaneous wound healing in diabetic rats by promoting endothelial function [79]. Moreover, EVs enhanced the proliferation, migration, and tube formation of vascular endothelial cells in vitro and even stimulated the production of FGF-1, VEGFA, VEGFR-2, Angiopoietin 1 (ANG-1), E-selectin, C-X-C Motif Chemokine Ligand 16 (CXCL-16), endothelial NOS (eNOS), and C-X-C motif chemokine ligand 8 (IL-8/CLCX8) in these cells. Those factors are important angiogenesis regulators. Wounds in the feet of diabetic rats had a significantly reduced ulcerated area when treated with EVs from adipose-derived MSCs overexpressing nuclear factor erythroid 2–related factor 2 (Nrf2), a basic leucine zipper (bZIP) protein that regulates the expression of antioxidant proteins. Increased granulation tissue formation, angiogenesis, and levels of growth factor expression, as well as reduced levels of inflammation and oxidative stress-related proteins, were detected in wound beds [80].

Genetically diabetic B6.Lepr ^db/db^ mice wounds treated with subcutaneous administration of EVs from human circulating fibrocytes (mesenchymal progenitor cells) were found to heal faster than controls [81]. This particular EV population was also capable of improving human diabetic skin cell migration in vitro, as well as enhancing the production of collagen I, III and alpha-smooth muscle actin (α-SMA). Moreover, HSP-90, STAT3, miR-21, miR124a, miR-125b, miR-126, miR-130a, and miR-132 were found to be part of this EV population’s cargo, similar to findings from other reports on stem cell EVs cargo [81]. HSP-90 is known to induce wound reepithelization, and STAT3 is a transcription factor that induces growth factor production. miR-124a and miR-125a could modulate inflammation; miR-21 induces collagen production; miR-126, miR-130a, and miR-132 are proangiogenic factors [81].

Genetically diabetic C57BLKS/J-Leprdb (db/db) mice wounds also decreased in size and showed improved vascularization after subcutaneous injection of pluripotent stem cells (iPSCs) EVs [82].

The use of biodegradable matrices for stem cell delivery to injured tissues is not a new concept. However, stem cell EVs associated with matrices for controlled and sustained delivery is still in early testing. Tao and collaborators proposed a system in which a chitosan hydrogel loaded with stem cell EVs (from synovium MSCs) overexpressing miR-126 accelerate wound healing in male Sprague–Dawley diabetic rats. In vitro results demonstrated that those miR-126-overexpressing EVs stimulated human dermal fibroblast proliferation and human dermal microvascular endothelial cell (HMEC-1) proliferation, migration, and tube formation [83].

## 5. Skin Rejuvenation

The promising capacities of stem cells in tissue repair also have raised questions about their ability to avoid aging damage. Stem cell-conditioned media from endothelial precursor cells differentiated from human embryonic stem cells have been used in skin rejuvenating research [84,85] with interesting results. The injection of conditioned media from those cells improved the aspect of skin wrinkles and skin aspect in women [84].

UV light damage and aging affect extracellular matrix collagen and elastin depots, both of which are key in the prevention of skin dehydration as well as in firmness and elasticity preservation. The beneficial effects of stem cell EVs for cellular matrix maintenance and collagen production as described previously could contribute to this effect, considering that vesicles are important components of stem cell-conditioned media [86,87].

Furthermore, reports have suggested that purified stem cell EVs could play a role in rejuvenating skin cells. A report from Oh and colleagues indicated that EVs from iPSCs could restore the function of aged human dermal fibroblasts. The authors reported that dermal fibroblasts pretreated with iPSC EVs resisted photoaging with UVB and did not overexpress matrix-degrading enzymes MMP-1/3 but, on the contrary, displayed a high expression of collagen I, as young fibroblasts do. The same effect was observed in senescent fibroblasts at 30th passage [88].

Kim and colleagues studied the capacity of human umbilical cord stem cell EVs to rejuvenate skin by modulating collagen production and permeation. They also investigated whether EVs acceptance could accelerate fibroblast proliferation. Not only did skin cells proliferate more after EVs endocytosis, but a better production of collagen and elastin in human skin models was also observed in their study [44].

Altogether, these studies indicate that stem cell EVs could be good candidates for therapeutic strategies against aging.

## 6. Angiogenesis

Angiogenesis is part of the wound healing proliferative phase, but it is also the result of a collection of intricate steps in itself, and it comprises a series of cellular events that lead to neovascularization [89].

Initially, the proliferation of endothelial cells to generate new capillaries is required, just as the proteolysis of the extracellular matrix. The latter is essential to achieve the invasion of endothelial cells into the stroma of the neighboring tissue. Lumen development follows as the new capillary sprout forms, and it reaches maturity as the basement membrane and adherent junction appear. Then, a new capillary channel is formed. Growth factors, cytokines, the plasminogen activator (PA) system, and MMPs are examples of important regulators of neovascularization [90,91,92].

Due to the extraordinary angiogenesis-stimulating capacity of stem cell EVs, there is a variety of tissues in which their potential was tested. We here focus on studies indicating that stem cell EVs could induce angiogenesis in generally accepted models.

Normal angiogenesis is essential for normal wound closure, while its absence is one of the hallmarks of human chronic wounds. Various stem cells types and their EVs can induce angiogenesis, including adipose, bone marrow, and umbilical cord MSCs, endothelial progenitor cells, and iPSCs [93]. Studies that focus on the capacity of EVs to induce angiogenesis have used different in vitro and in vivo approaches. Endothelial cell lines such as human umbilical vein endothelial cells (HUVECs) are commonly used for the assessment of endothelial proliferation and tube formation assays, respectively [94,95,96].

In an elegant study by Gong and colleagues, EVs from the MSC line C3H10T1/2 were capable of promoting HUVEC tube-like structure formation in vitro. Also, EVs were able to increase the mobilization of endothelial cells into a Matrigel plug subcutaneously transplanted into C57BL6 mice. The authors reported that there was increased blood flow inside the Matrigel plug as well [96].

Primary human, mouse, rat, and even equine stem cells generate EV populations that convey similar results. Adipose-derived MSCs-EVs were able to induce tube formation in HUVECs and HMECs in vitro [38,97]. When stem cells are exposed to PDGF or endothelial differentiation media, their production of EVs increases and their angiogenesis potential rises. Further studies on EVs cargo for this particular effect suggests that PDGF and miR-31 might be relevant for the observed effects, as PDGF in a known growth factor that promotes vascularization and miR-31 targets *HIF-1*, a gene that is antiangiogenic in HUVECs. In vivo studies revealed that EVs could induce microvessel outgrowth of mouse aortic rings as well as the vascular formation of mouse Matrigel plugs. miR-125a is also part of adipose stem cell EVs cargo with angiogenic potential as it targets angiogenic inhibitor delta-like 4 (DLL4) [96]. Furthermore, experimental evidence indicated that a CD34-expressing fraction of EVs from human MSCs is one of the main contributors to improved angiogenesis in a mouse ischemic hindlimb model. The reason for this is their high expression of miR-126-3p, a well-known promoter of vascularization. MicroRNA miR-126-3p targets sprouty-related EVH1 domain-containing protein 1 (*SPRED1*), which downregulates *VEGF*, *ANG1*, *ANG2*, *MMP9*, and thrombospondin 1 (*THBS1*) [37].

As previously mentioned, the microenvironment in which stem cells are cultivated affects their EVs cargo directly. Low oxygen concentration, for example, affects stem cells in important ways. Hypoxia is known as a stimulus for cytokine production that culminates in the improved angiogenic potential of EVs and is even reported to increase EVs release [98]. Human adipose-derived MSCs subjected to this condition release EVs that activate the PKA pathway and improve angiogenesis [99] and human mesenchymal stem cells submitted to hypoxia release EVs enriched with miR-26a, which in turn targets GSK3β, another potent antiangiogenic factor. Furthermore, EVs were found to reverse reductions in Wnt1 and β-catenin levels caused by cardiac tissue infarction [100]. Low oxygen concentration also increases human dental pulp MSCs-EVs’ concentration of Jagged1, which activates Notch and improves the capacity of EVs to induce capillary-like tube formation in HUVECs [98]. It is interesting to note that nitric oxide, as well as oxygen, could improve stem cells’ capacity of inducing vascular production. MicroRNA miR-126 is overexpressed in these vesicles; thus, they promote better vasculogenesis [101].

Human umbilical cord stem cell EVs were shown to carry mRNAs for growth factors that induce angiogenesis, such as *FGF*, *VEGF*, *TGFB1*, *HGF*, *CTGF*, and *IL-6*, and they also induced tube formation in HUVEC cells [40,102]. miR-210, enriched in human and mouse bone marrow EVs, targets ephrin A3 (Efna3), a known downregulator of angiogenesis [103]. Mouse bone marrow stem cell EVs also carry VEGF, VEGFR1, and VEGFR2, and activate the SRC, AKT, and ERK pathways, which are all relevant for angiogenesis in endothelial cells [104,105]. Human bone marrow stem cell EVs carry an extracellular matrix metalloproteinase inducer (EMMPRIN) that, once internalized, activates the ERK pathway. When incorporated into endothelial cells, placenta stem cell EVs stimulate both endothelial tube formation and migration, as well as enhanced angiogenesis-related gene expression. Laser Doppler blood flow analysis showed that vesicles infusion also enhanced angiogenesis in an in vivo murine auricle ischemic injury model [52].

## 7. Limitations of Stem Cell EVs Use

While there has been improvement in the knowledge about EVs effectiveness and mechanisms of tissue repair, it is important to consider the limitations for their use. The development of pharmaceutical applications must rely not only on effectiveness but also on the safety of a given formulation. Previously, it was demonstrated that formulations based on EVs were safe for use [106].

One advantage of stem cell EVs in therapy is their capacity to stimulate beneficial effects without generating rejection, as allogenic stem cell transplants occasionally do. Still, various reports suggest that part of the stem cell EVs cargo, especially some microRNAs, could cause undesirable effects, such as tumorigenesis [107,108]. The biology of microRNA is complex, and a given molecule of this particular class could provoke distinct and even opposite effects in different tissues [109,110]. Hence, biosafety studies are imperative when using natural or tailored stem cell EVs. Moreover, these tests should consider the intended administration method and the biodistribution of EVs.

EVs biodistribution studies are essential to avoid undesired effects caused by the misallocation of vesicles at sites other than the intended target after administration. Currently, the majority of work in this field addresses a variety of vesicles from different origins administrated intravenously. Researchers use labeling methods such as bioluminescence to this end [111].

Evidence suggests that, once injected, stem cell EVs preferably target the liver, spleen, and lungs, but efforts are also underway to create strategies that would allow specific targeting in other tissues [111]. Since stem cell EVs for skin repair can be administrated intradermally, subcutaneously, or topically, further investigation is imperative. A report indicated that superparamagnetic iron oxide nanoparticles for magnetic resonance tracking were used to label melanoma EVs injected in C57BL/6 mouse-pad foot (intradermal/subcutaneous). Those were then followed by standard magnetic resonance imaging approaches and the results indicated that the labeled EVs reached the animals’ lymph nodes [112]. This type of technique could be used for stem cell EVs tracking, for example.

Another important issue that should be considered when working with EVs is the poor yield that is usually obtained from conditioned culture media as opposed to the high amounts required for therapy. Stem cell EVs scale production must be feasible for them to become viable for clinical use. The solution would likely involve the use of bioreactors. In a hollow fiber bioreactor, the EV yield achieved was found to be, in milligrams, approximately 10-fold greater than cultures that grown in T-flasks and cell factories, while simultaneously resulting in a higher concentration/mL conditioned medium [113].

Stem cell EVs homogenous populations and cargo are a challenge to obtain, and this premise makes their use in therapeutics more difficult. Even optimized methods of EVs purification that associate ultracentrifugation and filtration or an immuno-affinity step that targets membrane proteins CD81, CD9, and CD63 have not achieved ideal purity. The solution could be to invest in tailored synthetic EVs with uniform lipidomic, proteomic, and transcriptomic content [114,115,116].

## 8. Concluding Remarks

Stem cells EVs seem promising as a tool for the so-called “cell therapy without cells” due to their effects in proliferation, migration, rejuvenation, inflammation, and scarring control in epithelial tissues. Potential caveats born from their lack of uniformity or low yields from conditioned media could be avoided by designing tailored EVs with the desired cargo for each pathology of interest.

## Figures and Tables

**Table 1:** Stem cell EVs’ cargo. This table summarizes relevant findings from different authors on EVs content.

Vesicle Type According to Authors	Source (Stem Cell Type)	Technique to Assess Cargo Content	Most Relevant Findings of Cargo Content	Reference
Exosomes	Human CD34^+^ stem cells	MicroRNA array, angiogenic protein array	miR-126-3p was enriched in exosomes and promoted angiogenesis.	Mathiyalagan et al., 2017 [37]
EVs	Adipose stem cells stimulated or not with platelet-derived growth factor (PDGF)	Protein array for 507 proteins	Adipose-derived stem cell EVs Angiogenic factors: angiopoietin-like factor, APJ, IL-1α, MIP 2. Anti-angiogenic factors: angiostatin, endostatin, polyvalent regulators, activin C, granulocyte-colony stimulating factor (GCSF). PDGF adipose-derived mesenchymal stem/stromal cell (MSCs) EVs Angiogenic factors: thrombopoietin, matrix metalloproteinases (MMPs), oncostatin M (OSM). Anti-angiogenic factors: chemokine (C-C motif) ligand 21 (6Ckine), TIMP metallopeptidase inhibitor 1 (TIMP-1), leukemia inhibitory factor (LIF).	Lopatina et al., 2014 [38]
Exosomes	Human bone marrow stem cells	HiRIEF LC-MS/MS proteome in the normoxic or peripheral arterial disease (PAD)-like microenvironment	A PAD-like microenvironment increases the expression of epidermal growth factor (EGF), fibroblast growth factor (FGF), and PDGF. Furthermore, a PAD-like microenvironment induces elevated exosome secretion and induces angiogenesis in vitro via the nuclear factor kappa-light-chain-enhancer of activated B cells (NF-kB).	Anderson et al., 2016 [39]
EVs	Cord blood stem cells	qRT-PCR array expression profiling (human mesenchymal stem cells RT2 profiler PCR array system)	Enriched messenger RNAs (mRNAs): connective tissue growth factor (CTGF/ CCN2), FGF, IL-6, transforming growth factor beta 1 (TGFB1), vascular endothelial growth factor (VEGF), hepatocyte growth factor (HGF).	Montemurro et al., 2016 [40]
EVs	Porcine adipose stem cells	LC-MS/MS proteomic profiling	EVs contained pro-inflammatory agents (complement system) and promoted angiogenesis, blood coagulation (vWF, coagulation factor X, and plasma kallikrein), matrix remodeling (matrix metallopeptidase 9-MMP9, TGFβ family), and apoptosis (netrin-1).	Eirin et al., 2016 [41]
EVs	Human umbilical cord and human bone marrow stem cells	LC-MS/MS proteomic profiling	797 proteins identified linked to immune response, phagocytosis, and innate immunity.	Angulski et al., 2017 [42]
Exosomes	Human umbilical cord stem cells	microRNA array	15 upregulated microRNAs compared to fibroblasts. The most relevant were: miR-21, miR-146a, and miR-181.	Ti et al., 2016 [43]
Exosomes	Human umbilical cord blood-derived mesenchymal stem cells	Human growth factor antibody array	Presence of various growth factors such as TGFb, HGF, bFGF, VEGF, EGF, PDGF, FGF, IGFBP6.	Kim et a., 2017 [44]
EVs	Bone marrow, gingiva, and skin stem cells	Cytokine array analysis	IL-1RA presence is higher in gingiva stem cell EVs and better controls inflammation.	Kou et al., 2018 [45]
